# Biatrial Inflammatory and Fibrotic Remodeling in Severe Aortic Stenosis Compared with CABG Controls

**DOI:** 10.3390/metabo16080574

**Published:** 2026-08-14

**Authors:** Adrian-Grigore Merce, Daniel Dumitru Nișulescu, Anca Hermenean, Adrian-Petru Merce, Raluca Muntean, Daniel-Miron Brie, Oana-Maria Burciu, Dan Găiță, Adina Ionac, Simina Crișan, Cristian Mornoș

**Affiliations:** 1 Doctoral School Medicine–Pharmacy, “Victor Babeș” University of Medicine and Pharmacy Timișoara, Efimie Murgu Sq. No. 2, 300041 Timișoara, Romania; adrian-grigore.merce@umft.ro (A.-G.M.);; 2 Department of Cardiology, Institute of Cardiovascular Diseases Timișoara, 300310 Timișoara, Romania; 3 Advanced Research Center, Institute for Cardiovascular Diseases Timișoara, 300310 Timișoara, Romania; 4 Multidisciplinary Doctoral School, “Vasile Goldiș” Western University of Arad, 310025 Arad, Romania; 5 Department of Histology, Faculty of Medicine, “Vasile Goldiș” Western University of Arad, 310025 Arad, Romania; 6 “Aurel Ardelean” Institute of Life Sciences, “Vasile Goldiș” Western University of Arad, 310025 Arad, Romania; 7 Department of Cardiovascular Surgery, Institute of Cardiovascular Diseases Timișoara, 300310 Timișoara, Romania; 8 Cardiology Department, “Victor Babeș” University of Medicine and Pharmacy Timișoara, Eftimie Murgu Sq. No. 2, 300041 Timișoara, Romania

**Keywords:** aortic stenosis, atrial fibrosis, atrial remodeling, inflammation, RT-qPCR, echocardiography, CABG controls

## Abstract

**Highlights:**

**What are the main findings?**

- Building on extensive evidence that aortic stenosis is a whole-heart fibro-remodeling disease, this study provides complementary direct human biatrial tissue evidence: severe aortic stenosis was associated with greater patient-level left- and right-atrial fibrosis than CABG controls without significant valvular disease.
- Pooled RT-qPCR outputs descriptively suggested a biatrial inflammatory/profibrotic profile involving IL-6, TNF-alpha, and TGF-beta; no inferential *p* values are reported for these group-level molecular endpoints.

**What are the implications of the main findings?**

- The findings complement the established ventricular fibrosis literature by extending direct tissue characterization to both atria rather than proposing fibrosis as a newly recognized aortic stenosis mechanism.
- Patient-level molecular analyses, formal laboratory reproducibility studies, and advanced atrial functional imaging are required to quantify individual mechanistic relationships.

**Abstract:**

Background: Extensive prior work has established severe aortic stenosis as a whole-heart remodeling process involving chronic pressure overload, concentric hypertrophy, myocardial stiffness, and progressive ventricular fibrosis. However, direct paired histological characterization of both atria remains limited. Methods: This single-center observational comparative study included 28 patients with severe aortic stenosis referred for surgical aortic valve replacement and 14 coronary artery bypass grafting (CABG) controls without significant valvular disease, consecutively enrolled between 9 May 2024 and 28 April 2025. Left- and right-atrial tissue samples were collected intraoperatively. The relative expression of interleukin-6 (IL-6), tumor necrosis factor-alpha (TNF-alpha), and transforming growth factor-beta (TGF-beta) was quantified by RT-qPCR using pooled tissue samples and interpreted descriptively as a group-level molecular output. Atrial fibrosis was measured histologically at the patient level using Masson’s trichrome staining and digital image analysis. Results: Compared with CABG controls, patients with severe aortic stenosis demonstrated greater left-atrial fibrosis (median: 19.12% [IQR: 16.12–23.12] vs. 11.54% [9.53–14.29]; *p* < 0.0001) and right-atrial fibrosis (16.33% [15.07–19.13] vs. 8.40% [5.75–11.27]; *p* = 0.0001). Pooled RT-qPCR outputs descriptively suggested higher IL-6, TNF-alpha, and TGF-beta expression in both atria, although biological replication was insufficient for inferential testing. Echocardiographically, the aortic stenosis group showed higher aortic jet velocity and transvalvular gradients, thicker ventricular walls, a larger right-ventricular diameter, higher pulmonary artery systolic pressure, and a larger estimated left-atrial volume. A sensitivity analysis of 13 within-group correlations found that no correlation retained statistical significance at q < 0.05. Exploratory regressions restricted to patient-level histological outcomes suggested that severe aortic stenosis status remained associated with left- and right-atrial fibrosis after limited adjustment for age and sex. Conclusions: These findings complement the extensive ventricular fibrosis literature by providing paired human biatrial tissue evidence of greater atrial fibrosis in severe aortic stenosis compared with CABG controls. The pooled molecular findings remain descriptive and require validation using patient-level biological measurements.

## 1. Introduction

Calcific aortic stenosis is the most common valvular heart disease requiring intervention in Europe and North America and is associated with substantial cardiovascular morbidity and mortality once symptoms or ventricular decompensation develop [1,2,3,4,5]. Rather than representing a passive degenerative lesion confined to the valve, aortic stenosis is currently recognized as an active fibrocalcific and whole-heart remodeling process involving lipid infiltration, inflammation, osteogenic differentiation, extracellular-matrix turnover, progressive valvular calcification, and secondary myocardial injury [4,5,6,7,8,9,10,11,12,13,14,15,16,17,18]. Chronic left-ventricular pressure overload initially promotes compensatory concentric hypertrophy but may subsequently lead to diffuse interstitial and replacement fibrosis, increased myocardial stiffness, diastolic dysfunction, impaired contractile reserve, and clinical decompensation [10,11,12,13,14,15,16,17,18]. Accordingly, myocardial fibrosis is an established component of the pathophysiology and prognostic assessment of severe aortic stenosis.

The consequences of this remodeling process extend beyond the left ventricle. Sustained elevations in left-ventricular filling pressure increase left-atrial wall stress and promote atrial dilatation, impaired reservoir, conduit, and booster-pump function, extracellular-matrix remodeling, and susceptibility to atrial fibrillation [19,20,21,22,23,24,25,26,27,28,29,30,31,32]. Left-atrial enlargement and functional impairment have been associated with disease severity and adverse outcomes, while left-atrial strain may provide prognostic information beyond conventional chamber dimensions. Right-atrial remodeling may also occur through pulmonary vascular and right-sided hemodynamic involvement, although it has been less extensively characterized. Most available studies have focused on ventricular fibrosis, imaging-derived atrial remodeling, or isolated left-atrial measurements. Direct patient-level histological assessment of paired left- and right-atrial tissue within the same severe aortic stenosis cohort remains limited [22,23,30,31,32,33,34,35].

Inflammatory, profibrotic, oxidative, and metabolic pathways may contribute to the atrial component of this remodeling process. Interleukin-6, tumor necrosis factor-alpha, and transforming growth factor-beta participate in immune-cell recruitment, fibroblast activation, extracellular-matrix deposition, and the development of atrial and valvular fibrosis [24,25,26,27,28,29]. Recent human and translational evidence has further linked altered myocardial sodium–glucose cotransporter 2 expression, redox-modified proteins, systemic inflammatory biomarkers, and transcriptomic pathways involving immune activation, cellular metabolism, and TGF-beta signaling to the severity and clinical expression of aortic stenosis [36,37,38,39,40]. These observations support an intersection between hemodynamic stress, inflammation, oxidative imbalance, metabolism, and tissue fibrosis. However, molecular results obtained from composite tissue pools cannot establish patient-level pathway activation or identify individual molecular determinants and must therefore be interpreted descriptively.

Against this background, the present study evaluated paired left- and right-atrial tissue obtained from patients undergoing surgical aortic valve replacement for severe aortic stenosis and from patients undergoing isolated coronary artery bypass grafting without significant valvular disease. The CABG group was selected as a clinically relevant surgical comparator that permitted intraoperative atrial sampling in the absence of significant valvular obstruction; however, it represents a population with established ischemic cardiovascular disease rather than a healthy control group. We compared patient-level histologically quantified atrial fibrosis and echocardiographic remodeling between the two groups and descriptively assessed pooled atrial expression of IL-6, TNF-alpha, and TGF-beta. We hypothesized that severe aortic stenosis would be associated with greater left- and right-atrial fibrosis and a more pronounced pressure-overload remodeling phenotype than the CABG comparator group. The study was designed to provide complementary direct evidence of biatrial tissue involvement within the established whole-heart remodeling framework of aortic stenosis, rather than to propose fibrosis as a newly recognized disease mechanism.

## 2. Materials and Methods

### 2.1. Study Design and Patient Population

**Figure 1:**
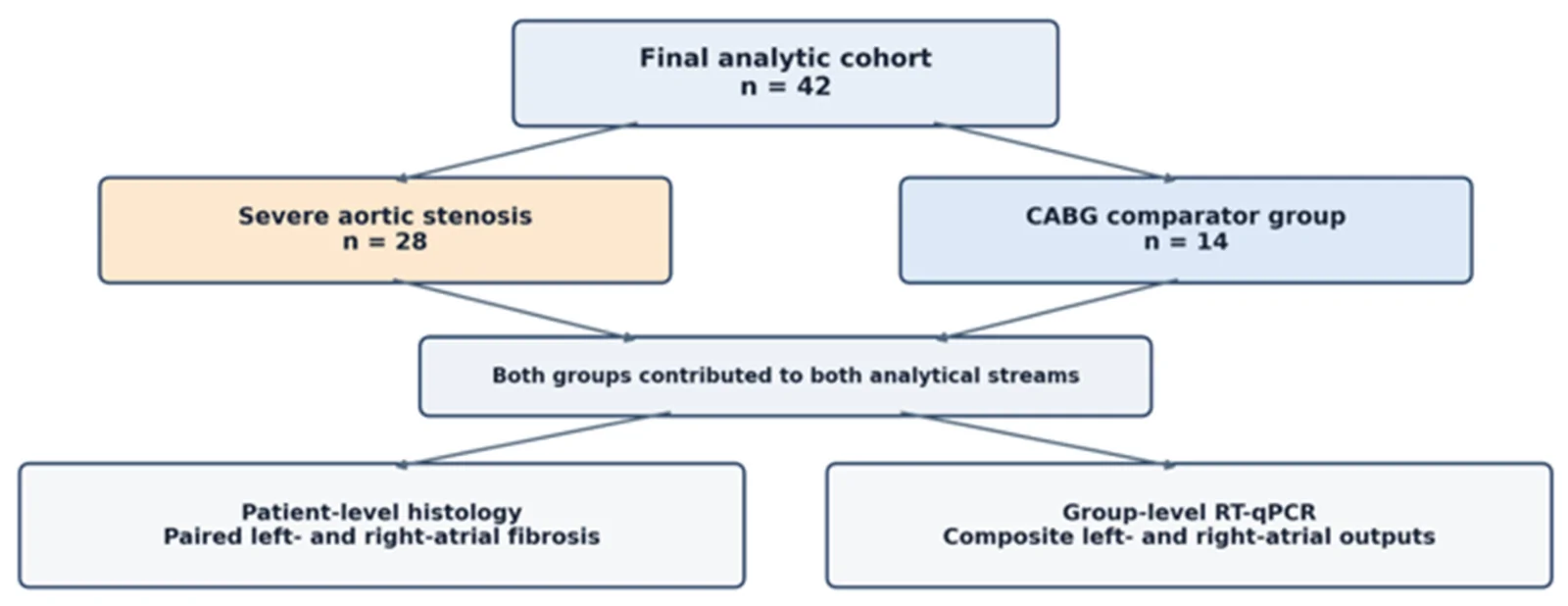
Available study cohort and analytical workflow.

This was a single-center, observational, cross-sectional comparative study of adults undergoing elective cardiac surgery at the Institute of Cardiovascular Diseases, Timisoara, Romania. Patients were consecutively enrolled between 9 May 2024 and 28 April 2025. Eligible participants underwent either surgical aortic valve replacement for guideline-defined severe aortic stenosis or isolated CABG for atherosclerotic coronary artery disease without significant valvular pathology, provided written consent, and had atrial tissue adequate for histological analysis. Emergency procedures, absence of consent, significant valvular disease in the CABG comparator group, or inadequate tissue for the prespecified analysis precluded inclusion. The final analytic population comprised 42 patients: 28 with severe aortic stenosis and 14 CABG controls. A prospective log of all patients assessed before consent or tissue feasibility was not retained; therefore, pre-enrollment screening and exclusion counts cannot be reconstructed reliably. The available cohort and analytical data structure are summarized in Figure 1.

### 2.2. Tissue Sampling and Processing

Left- and right-atrial tissue samples were collected intraoperatively under sterile conditions whenever technically feasible and safe. Tissue fragments were subdivided for molecular and histologic analyses. One portion was stored in RNAlater and another snap-frozen in liquid nitrogen for downstream RNA work, whereas formalin-fixed paraffin-embedded samples were used for histologic assessment. Tissue processing and downstream laboratory analyses were performed at Vasile Goldis Western University of Arad, within the Aurel Ardelean Institute of Life Sciences, following the standardized laboratory workflow described below and in the reference tissue-processing manuscript.

### 2.3. RT-qPCR Analysis

Total RNA was isolated from atrial tissue using the Direct-zol RNA MiniPrep Plus kit (Zymo Research, Irvine, CA, USA; R2072) according to the manufacturer’s instructions. Samples were combined before RNA extraction into one composite biological pool for each study-group/atrial-side combination (severe AS-left atrium, severe AS-right atrium, CABG-left atrium, and CABG-right atrium). RNA yield and purity were checked at the pooled-extract level before reverse transcription; individual-patient RNA-quality metrics were therefore not available. Complementary DNA was synthesized using the High-Capacity cDNA Reverse Transcription Kit (Applied Biosystems, Irvine, CA, USA; 4368814). Quantitative real-time PCR for IL-6, TNF-alpha, and TGF-beta was performed with SYBR Green Master Mix (Applied Biosystems, Irvine, CA, USA; 4309155) on a StepOnePlus system. Reactions were run in technical replicates to assess assay precision. GAPDH was selected a priori as the reference gene in accordance with the established laboratory workflow, and relative expression was calculated by the 2^-DeltaDeltaCt^ method. A formal comparison of GAPDH stability against 18S rRNA or a multi-gene housekeeping panel was not performed. Because the dataset contained one composite biological pool per group and atrial side, technical replicates were not treated as independent biological observations; molecular outputs are presented descriptively without standard deviations, confidence intervals, or inferential *p* values and were not used in patient-level correlations or regressions.

### 2.4. Histology and Fibrosis Quantification

Formalin-fixed paraffin-embedded atrial samples were sectioned at 4–5 micrometers and stained with Masson’s trichrome. Digital images were acquired under standardized magnification and exposure settings. Whenever adequate tissue was available, five non-overlapping fields were selected using a systematic random approach across viable myocardium; folds, tears, cautery artifact, and large vessels were avoided. ImageJ version 1.54g, color thresholds were defined before batch quantification on representative stained sections and then held constant within the analytical batch. Fibrosis was expressed as collagen-positive area divided by total viable tissue area and quantified separately in the left and right atria at the patient level. Quantification was performed by a single trained observer. A formal second-reader or repeat-reader reproducibility study was not performed, and diagnostic blinding was not prospectively documented.

### 2.5. Clinical, Laboratory, and Echocardiographic Assessment

Demographic data, comorbidities, chronic medication, prior myocardial infarction, atrial fibrillation, and perioperative laboratory data were extracted from hospital records. Previous catheter or surgical ablation was not systematically captured. Preoperative transthoracic echocardiography provided chamber dimensions, left-ventricular diameters and volumes, ejection fraction, transmitral filling, aortic valve hemodynamics, pulmonary pressures, right-ventricular dimensions, and categorical grading of valvular lesions. Dedicated left-atrial strain and the minimum and pre-atrial-contraction volumes required for reservoir, conduit, and booster-pump function were not available and could not be reconstructed retrospectively.

### 2.6. Ethics Statement

The study was conducted in accordance with the Declaration of Helsinki and local institutional requirements for studies involving human surgical tissue. The doctoral research protocol entitled ‘Thrombospondins and their impact on cardiac remodeling in atrial myopathy’ received ethical approval from the Ethics Committee for Scientific Research of the Victor Babes University of Medicine and Pharmacy Timisoara (approval No. 49/02.10.2023), the Ethics Committee for Research and Development of the Institute of Cardiovascular Diseases Timisoara (approval No. 16027/20.12.2023), and the Ethics Committee for Scientific Research of Vasile Goldis Western University of Arad (approval No. 14/26.03.2024). The approved study sites included the Victor Babes University of Medicine and Pharmacy Timisoara, the Institute of Cardiovascular Diseases Timisoara, the Aurel Ardelean Institute of Life Sciences within Vasile Goldis Western University of Arad, and the Faculty of Biology. All participants provided written informed consent before inclusion and tissue sampling.

### 2.7. Statistical Analysis

Statistical analyses were performed using MedCalc Statistical Software, version 23.6.0 (MedCalc Software Ltd., Ostend, Belgium). No a priori sample-size calculation was performed; the sample was determined by consecutive surgical recruitment and availability of adequate atrial tissue during the prespecified study period. The study was therefore expected to identify large between-group histological differences but was not designed to exclude moderate associations or support highly parameterized multivariable models. Categorical variables are reported as counts and percentages and were compared using the chi-square or Fisher exact test. Continuous echocardiographic and laboratory variables are reported as mean plus or minus standard deviation and were compared using Welch’s *t* test. Patient-level fibrosis is reported primarily as median [interquartile range], with mean plus or minus standard deviation supplied secondarily, and was compared using the Mann–Whitney U test. Pooled RT-qPCR fold changes are descriptive group-level outputs; technical replicates were not analyzed as biological replicates and no molecular inferential *p* values were calculated. Within the severe AS group, exploratory correlations were restricted to patient-level histological and echocardiographic variables and used Spearman’s coefficient. Reported *p* values are nominal. As a multiplicity sensitivity analysis, Benjamini–Hochberg false-discovery-rate adjustment was applied across the 13 correlation tests; neither nominal association retained q < 0.05. Whole-cohort linear regressions evaluated severe AS status in relation to left- and right-atrial fibrosis with limited adjustment for age and sex. Additional covariates were not entered simultaneously because the cohort contained only 42 patients and several group-associated variables showed sparse cells or near separation, creating substantial overfitting risk. These models do not eliminate confounding by ejection fraction, prior myocardial infarction, atrial fibrillation, antiplatelet or anticoagulant therapy, or SGLT2 inhibitor use and are interpreted as exploratory associations rather than independent causal effects.

## 3. Results

### 3.1. Clinical Characteristics and Treatment Profile

The final analytic cohort included 28 patients with severe aortic stenosis and 14 CABG controls without significant valvular disease (Figure 1). Age, sex distribution, residence, hypertension, chronic kidney disease, diabetes, and obesity were broadly comparable. A preserved EF phenotype was more frequent in the aortic stenosis group, whereas reduced EF and previous myocardial infarction were more common among CABG controls. Anticoagulant and vitamin K antagonist use were more frequent in severe AS, while clopidogrel and SGLT2 inhibitor use were more common in controls. These imbalances are potential confounders and are not resolved by the limited age- and sex-adjusted models. All the clinical characteristics are presented in Table 1.

### 3.2. Echocardiographic Findings

**Figure 2:**
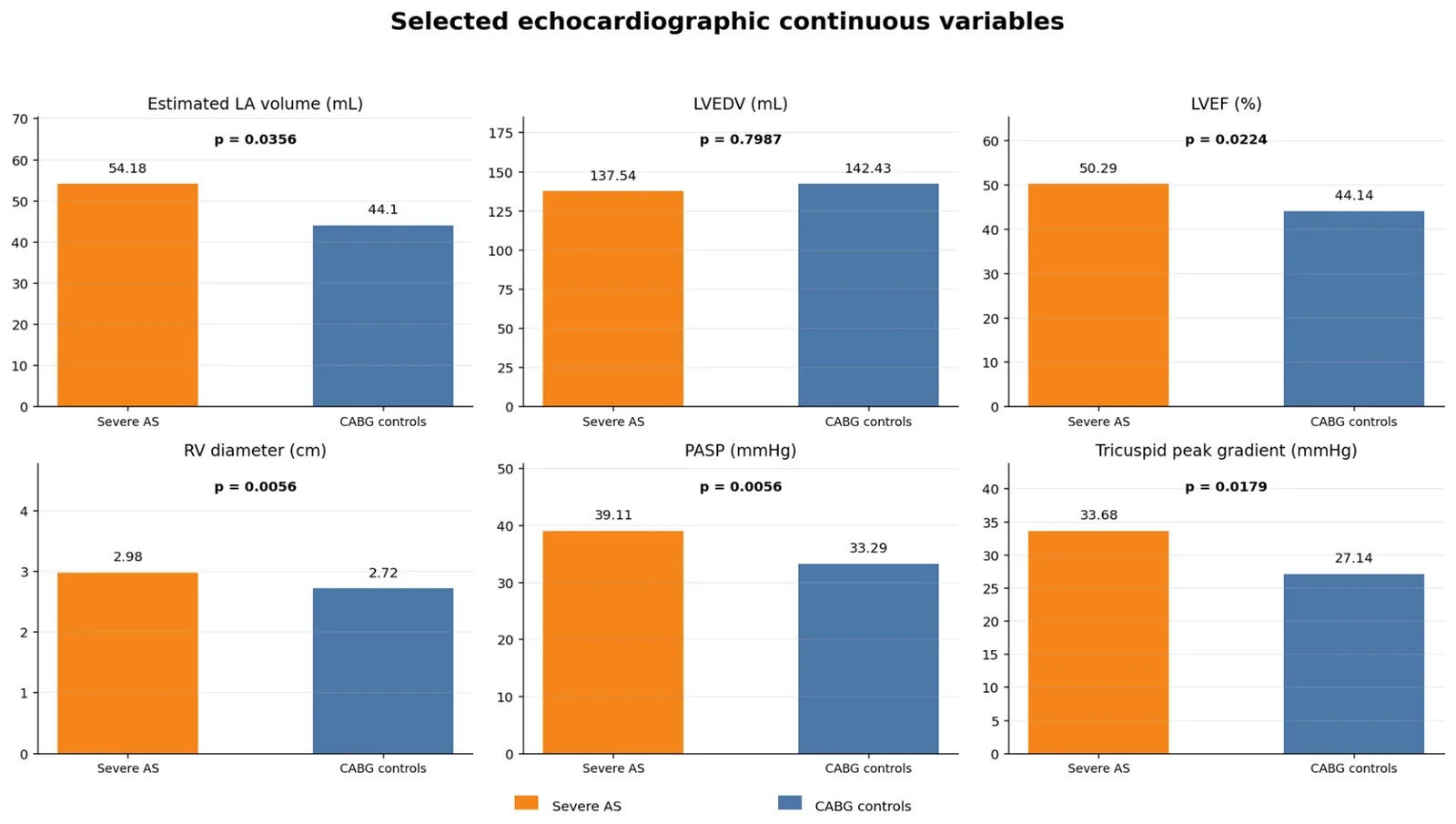
Selected echocardiographic continuous variables.

Echocardiographic evaluation showed the expected differences in aortic valve hemodynamics and pressure-overload remodeling. All severe AS patients had guideline-defined severe stenosis, whereas CABG controls had no significant valvular obstruction. Severe AS was associated with more frequent left-atrial dilation greater than 4.5 cm, higher aortic Vmax and transvalvular gradients, thicker interventricular septum and posterior wall, larger right-ventricular diameter, higher tricuspid gradient and pulmonary artery systolic pressure, and greater estimated left-atrial volume. LVEF was higher in severe AS, whereas LV volumes were not significantly different. Categorical and continuous findings are presented in Table 2 and Table 3 and selected continuous variables in Figure 2. Dedicated atrial strain and phasic-volume measures were not available. The biological and mechanical prosthesis variables in Table 2 describe the aortic prosthesis implanted during the index operation; they do not indicate prior prosthetic valves or redo surgery.

### 3.3. Tissue Biomarker Expression and Atrial Fibrosis

**Figure 3:**
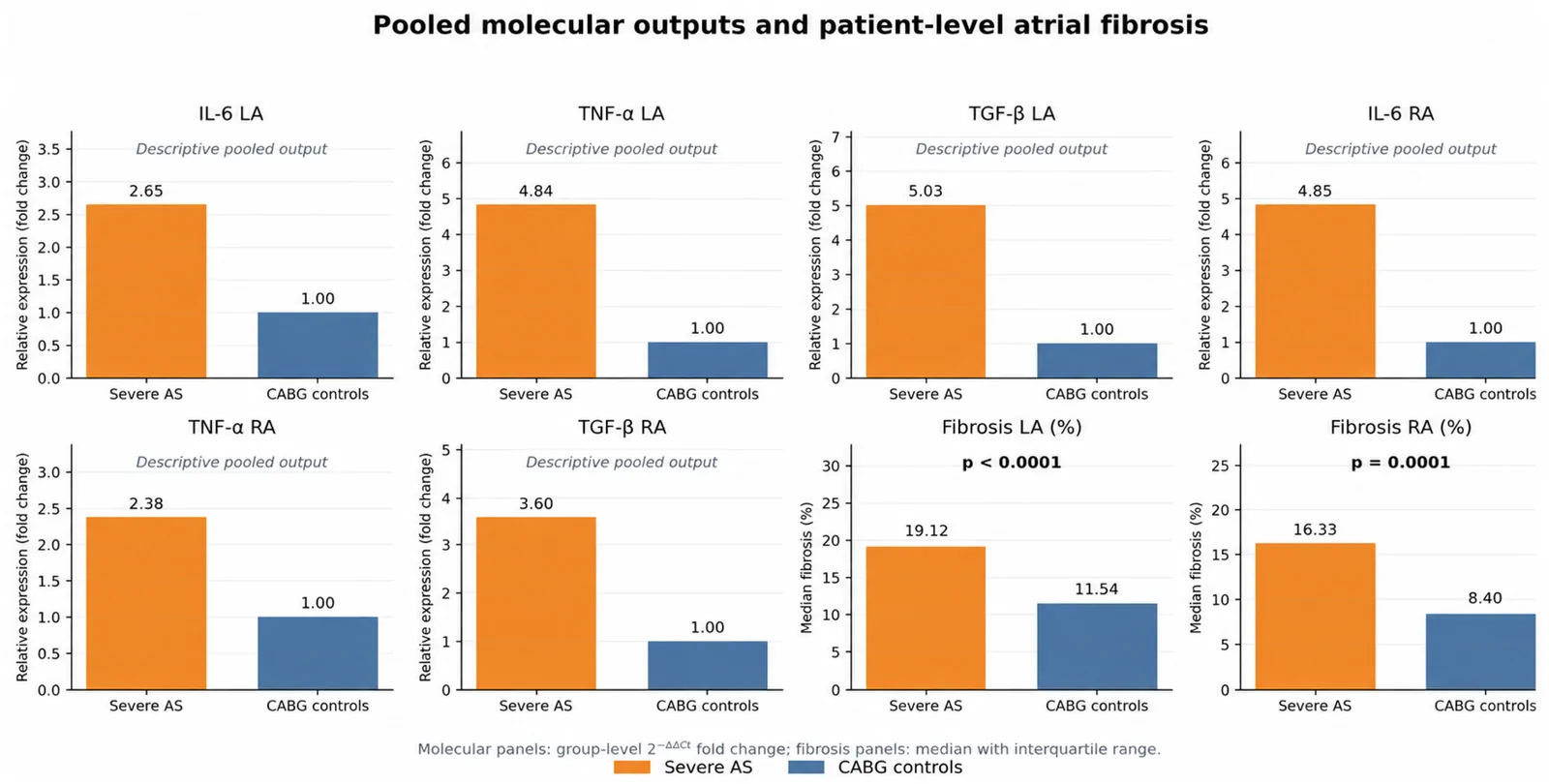
Descriptive pooled RT-qPCR fold changes and patient-level atrial fibrosis. Molecular panels show group-level 2^-DeltaDeltaCt^ fold changes relative to the CABG calibrator (1.00), without inferential *p* values. Fibrosis panels show median with interquartile range; *p* values are from Mann–Whitney U tests.

Pooled RT-qPCR outputs descriptively suggested higher IL-6, TNF-alpha, and TGF-beta expression in both atria of severe AS patients relative to the CABG calibrator. Because each study-group/atrial-side result originated from a single composite biological pool, no standard deviations, confidence intervals, or inferential *p* values are reported for molecular markers. Patient-level histology showed greater fibrosis in severe AS: left-atrial fibrosis was 19.12% [IQR: 16.12–23.12] versus 11.54% [9.53–14.29] (*p* < 0.0001), and right-atrial fibrosis was 16.33% [15.07–19.13] versus 8.40% [5.75–11.27] (*p* = 0.0001). Mean plus or minus standard deviation values are retained secondarily in Table 4. The corrected separation between descriptive molecular outputs and patient-level histological inference is shown in Table 4 and Figure 3.

### 3.4. Correlation Analysis

Correlation analysis within the severe aortic stenosis group was restricted to patient-level histological and echocardiographic variables. Left-atrial fibrosis did not show a nominal association with the tested parameters. Right-atrial fibrosis showed nominal correlations with estimated left-atrial volume (rho = 0.395, *p* = 0.0373) and ascending aortic diameter (rho = −0.424, *p* = 0.0246). The direct association between left- and right-atrial fibrosis was positive but non-significant (rho = 0.296, *p* = 0.1257). After Benjamini–Hochberg adjustment across all 13 correlations, neither nominal association remained significant (q = 0.242 for both). Given n = 28, negative findings and moderate effect estimates should be interpreted cautiously because the analysis was underpowered for modest correlations (Table 5).

### 3.5. Exploratory Regression Analysis

Exploratory whole-cohort regressions tested whether severe aortic stenosis status was associated with patient-level atrial fibrosis after limited adjustment for age and sex. Severe AS status was associated with higher left-atrial fibrosis (B = 8.548, 95% CI: 4.440 to 12.655, *p* = 0.0001, R2 = 0.329) and higher right-atrial fibrosis (B = 7.746, 95% CI: 4.396 to 11.097, *p* < 0.0001, R2 = 0.386). Molecular markers were not included because RT-qPCR lacked patient-level biological replication. The small cohort did not support simultaneous adjustment for the clinically important imbalances in LVEF, prior myocardial infarction, atrial fibrillation, antiplatelet or anticoagulant therapy, and SGLT2 inhibitor use. Accordingly, the models estimate limited adjusted associations and should not be interpreted as proof of an independent causal effect (Table 6).

## 4. Discussion

Within the context of extensive prior work on valvular and ventricular fibrosis in aortic stenosis, this study adds anatomically complementary evidence from paired human left- and right-atrial tissue. The main findings were greater patient-level biatrial fibrosis in severe AS than in CABG controls, an echocardiographic profile consistent with pressure-overload remodeling, and descriptive pooled evidence of IL-6-, TNF-alpha-, and TGF-beta-related pathway activation. These results should not be interpreted as establishing fibrosis as a new aortic stenosis hypothesis; their contribution is the direct biatrial histological comparison and the explicit separation of patient-level histological inference from pooled molecular description.

Aortic stenosis imposes chronic pressure overload on the left ventricle and indirectly on the left atrium. The transition from adaptive hypertrophy to extracellular-matrix expansion, stiffness, and decompensation has been demonstrated extensively by histology, cardiac magnetic resonance, and outcome studies [10,11,12,13,14,15,16,17,18]. Our higher transvalvular gradients and wall thicknesses are consistent with this established whole-heart framework rather than evidence for a previously unrecognized process. The larger estimated left-atrial volume similarly accords with prior work on chronic diastolic burden and prognosis [36,37,38,39]. Recent left-atrial strain studies further show that reservoir and phasic dysfunction can provide prognostic information beyond chamber size [40]; such measures were unavailable in our dataset.

The tissue findings extend the established hemodynamic framework to both atria. TGF-beta signaling, TNF-alpha/NF-kappaB activation, IL-6-related inflammation, oxidative stress, fibroblast activation, and extracellular-matrix dysregulation are recognized contributors to atrial and valvular remodeling [24,25,26,27,28,29]. Recent translational evidence is concordant: altered myocardial SGLT2 expression has been linked to inflammation, oxidative stress, and fibrosis in severe AS; redox-modified albumin can accelerate interstitial-cell calcification; systemic inflammatory indices carry prognostic information; and large human transcriptomic studies implicate immune, metabolic, and TGF-beta-related pathways in disease severity [36,37,38,39,40]. These data support a mechanistic intersection between metabolism, inflammation, oxidative stress, and fibrosis. Nevertheless, our qPCR results derive from four composite pools and cannot demonstrate patient-level pathway coupling or identify an individual molecular driver.

Within the severe AS group, patient-level histology did not demonstrate a robust relationship between atrial fibrosis and the measured echocardiographic variables. The two nominal right-atrial correlations did not survive Benjamini–Hochberg adjustment, and the direct left-to-right fibrosis correlation was non-significant. These findings may reflect limited power, measurement imprecision, chamber-specific load, or true biological heterogeneity. They should therefore be viewed as descriptive effect estimates rather than confirmatory associations. Larger cohorts incorporating individual-level RNA or protein measurements, left-atrial reservoir/conduit/booster-pump function, atrial strain, and CMR tissue characterization are required to test whether biatrial histological remodeling has reproducible functional and molecular correlates.

The comparator profile underscores the potential for residual confounding. CABG controls had more previous myocardial infarction, lower LVEF, and greater use of clopidogrel and SGLT2 inhibitors, whereas severe AS patients more often received vitamin K antagonists and other anticoagulant therapy. SGLT2 inhibition may attenuate inflammatory and oxidative pathways, while ischemic injury, antiplatelet treatment, and anticoagulant exposure may also modify tissue or circulating inflammatory signals. Because molecular measurements were pooled, these effects could not be adjusted at the patient level. The CABG group is therefore a clinically relevant surgical comparator, not a healthy control, and coronary disease itself can promote inflammation and fibrosis. The observed differences describe two real-world surgical phenotypes and cannot be attributed exclusively to valvular obstruction.

Several limitations warrant emphasis. First, this was a single-center feasibility study without an a priori sample-size calculation; it was suited to detecting large histological differences but underpowered for moderate correlations and expanded multivariable adjustment. A complete prospective screening log was not retained, limiting reconstruction of pre-enrollment exclusions. Second, RT-qPCR used one composite biological pool per group and atrial side. Technical replicates cannot substitute for biological replication, patient-level RNA-quality metrics were unavailable, and GAPDH was not compared with 18S rRNA or a multi-gene stability panel. Molecular findings are therefore descriptive only. Third, histology was quantified by one observer; formal diagnostic blinding and inter- or intraobserver reproducibility were not established. Fourth, residual confounding is likely because the groups differed in LVEF, prior myocardial infarction, antiplatelet and anticoagulant therapy, and SGLT2 inhibitor use; age- and sex-adjusted models do not resolve these imbalances. Atrial fibrillation may also influence fibrosis despite its non-significant between-group difference, and prior ablation history was not systematically captured. Fifth, CABG patients had ischemic heart disease and cannot be considered healthy controls. Finally, atrial strain, phasic atrial volumes, cardiac magnetic resonance, extracellular volume, and late gadolinium enhancement were unavailable. The two nominal correlations did not survive false-discovery-rate adjustment, reinforcing the exploratory nature of all secondary analyses.

## 5. Conclusions

This study complements the extensive literature on whole-heart fibrosis in aortic stenosis by providing paired human left- and right-atrial histological evidence. Severe aortic stenosis was associated with greater patient-level biatrial fibrosis and a pressure-overload echocardiographic phenotype than a CABG surgical comparator group. Pooled IL-6, TNF-alpha, and TGF-beta outputs were directionally consistent with inflammatory/profibrotic activation but remained descriptive because patient-level biological replication was unavailable. Clinically, the results support considering atrial remodeling as part of the broader cardiac response to severe aortic stenosis, while individual molecular mechanisms, functional atrial consequences, and independence from treatment and ischemic confounding require prospective validation.

## Figures and Tables

**Table 1:** Clinical characteristics, comorbidities, and treatment profile of the severe aortic stenosis and CABG control groups.

Variable	Severe AS (n = 28)	Control (n = 14)	*p* Value
Age, years	64.6 ± 8.7	62.4 ± 7.0	0.3812
Male sex	18 (64.3%)	11 (78.6%)	0.4852
Urban residence	15 (53.6%)	7 (50.0%)	1.0000
Persistent atrial fibrillation	4 (14.3%)	1 (7.1%)	0.6496
Postoperative atrial fibrillation	5 (17.9%)	4 (28.6%)	0.4508
Mixed dyslipidemia	19 (67.9%)	13 (92.9%)	0.1249
Hypertension	27 (96.4%)	13 (92.9%)	1.0000
Hypertension grade 1	0 (0.0%)	1 (7.1%)	0.3333
Hypertension grade 2	23 (82.1%)	11 (78.6%)	1.0000
Hypertension grade 3	5 (17.9%)	1 (7.1%)	0.6448
Chronic kidney disease	1 (3.6%)	0 (0.0%)	1.0000
Chronic obstructive pulmonary disease	2 (7.1%)	2 (14.3%)	0.5902
Diabetes mellitus	0 (0.0%)	0 (0.0%)	1.0000
Obesity	1 (3.6%)	0 (0.0%)	1.0000
Hyperuricemia	1 (3.6%)	0 (0.0%)	1.0000
Dilated cardiomyopathy	6 (21.4%)	5 (35.7%)	0.4588
Hypertensive/hypertrophic cardiomyopathy	13 (46.4%)	7 (50.0%)	1.0000
Preserved EF phenotype	23 (82.1%)	6 (42.9%)	0.0097
Reduced EF phenotype	5 (17.9%)	8 (57.1%)	0.0148
Previous myocardial infarction	0 (0.0%)	3 (21.4%)	0.0317
Anemia	6 (21.4%)	0 (0.0%)	0.0831
Vitamin K antagonist	17 (60.7%)	0 (0.0%)	0.0006
NOAC	1 (3.6%)	1 (7.1%)	1.0000
Beta-blocker	27 (96.4%)	14 (100.0%)	1.0000
Diuretic	27 (96.4%)	12 (85.7%)	0.2537
Spironolactone	27 (96.4%)	12 (85.7%)	0.2537
Statin	21 (75.0%)	13 (92.9%)	0.2328
Clopidogrel	0 (0.0%)	4 (28.6%)	0.0089
SGLT2 inhibitor	0 (0.0%)	3 (21.4%)	0.0317
Proton pump inhibitor	27 (96.4%)	13 (92.9%)	1.0000

**Table 2:** Categorical echocardiographic and valvular findings by study group.

Variable	Severe AS (n = 28)	Control (n = 14)	*p* Value
Left-atrial dilatation > 4.5 cm	22 (78.6%)	5 (35.7%)	0.0168
Atrial dilatation by estimated LA volume	25 (89.3%)	11 (78.6%)	0.3825
LVEDD > 5.7 cm	4 (14.3%)	5 (35.7%)	0.1326
LVEDV > 114.8 mL	18 (64.3%)	6 (42.9%)	0.1478
LVESV > 48.1 mL	19 (67.9%)	6 (42.9%)	0.1622
Diastolic dysfunction type I	24 (85.7%)	14 (100.0%)	0.2829
Diastolic dysfunction type II	4 (14.3%)	0 (0.0%)	0.2829
Severe aortic stenosis	28 (100.0%)	0 (0.0%)	<0.0001
Aortic regurgitation grade II	11 (39.3%)	2 (14.3%)	0.1587
Tricuspid regurgitation grade I	4 (14.3%)	6 (42.9%)	0.0592
Mild pulmonary hypertension	21 (75.0%)	14 (100.0%)	0.0752
Moderate pulmonary hypertension	6 (21.4%)	0 (0.0%)	0.0831
Biological aortic prosthesis implanted at index surgery	12 (42.9%)	0 (0.0%)	0.0034
Mechanical aortic prosthesis implanted at index surgery	16 (57.1%)	0 (0.0%)	0.0011
Fibrotic aortic valves	5 (17.9%)	10 (71.4%)	0.0021

**Table 3:** Continuous echocardiographic variables by study group.

Variable	Severe AS (n = 28)	Control (n = 14)	*p* Value
Ascending aorta, cm	3.87 ± 0.58	3.41 ± 0.41	0.0055
LVEDD, cm	5.00 ± 0.68	5.29 ± 0.78	0.2426
LVEDV, mL	137.54 ± 46.54	142.43 ± 62.75	0.7987
LVESV, mL	67.36 ± 26.27	82.00 ± 49.01	0.3106
LVEF, %	50.29 ± 5.48	44.14 ± 8.38	0.0224
Mitral E wave, m/s	0.75 ± 0.41	0.64 ± 0.21	0.2242
Mitral A wave, m/s	0.93 ± 0.25	0.95 ± 0.15	0.7294
E/A ratio	0.83 ± 0.44	0.66 ± 0.17	0.0881
Aortic Vmax, m/s	4.45 ± 0.99	1.15 ± 0.43	<0.0001
Aortic peak gradient, mmHg	82.07 ± 31.64	5.64 ± 6.34	<0.0001
Aortic mean gradient, mmHg	49.86 ± 20.66	2.93 ± 3.79	<0.0001
Tricuspid peak gradient, mmHg	33.68 ± 6.92	27.14 ± 8.23	0.0179
Pulmonary artery systolic pressure, mmHg	39.11 ± 7.59	33.29 ± 5.08	0.0056
IVS thickness, cm	1.63 ± 0.28	1.23 ± 0.18	<0.0001
Posterior wall thickness, cm	1.38 ± 0.17	1.18 ± 0.13	0.0003
Right-ventricular diameter, cm	2.98 ± 0.26	2.72 ± 0.26	0.0056
Estimated left-atrial volume, mL	54.18 ± 16.53	44.10 ± 12.65	0.0356

**Table 4:** Descriptive pooled molecular outputs and patient-level atrial fibrosis by study group.

Variable	Severe AS	CABG Controls	*p* Value
IL-6 LA	2.65-fold	1.00 (calibrator)	Not applicable
TNF-α LA	4.84-fold	1.00 (calibrator)	Not applicable
TGF-β LA	5.03-fold	1.00 (calibrator)	Not applicable
Fibrosis LA, %	19.12 [16.12–23.12]; 20.77 ± 6.52	11.54 [9.53–14.29]; 12.17 ± 4.56	<0.0001
IL-6 RA	4.85-fold	1.00 (calibrator)	Not applicable
TNF-α RA	2.38-fold	1.00 (calibrator)	Not applicable
TGF-β RA	3.60-fold	1.00 (calibrator)	Not applicable
Fibrosis RA, %	16.33 [15.07–19.13]; 16.47 ± 5.28	8.40 [5.75–11.27]; 9.01 ± 4.46	0.0001

Note: RT-qPCR entries are descriptive group-level 2^-DeltaDeltaCt^ fold changes from one composite biological pool per study-group/atrial-side combination; CABG controls serve as the calibrator (1.00). Technical replicate variability is not biological replication, and no molecular inferential tests were performed. Fibrosis is reported primarily as median [interquartile range], with mean plus or minus standard deviation in parentheses; fibrosis *p* values are from Mann–Whitney U tests.

**Table 5:** Patient-level correlations between histological atrial fibrosis and echocardiographic remodeling parameters within the severe aortic stenosis group.

Variable 1	Variable 2	Interpretation	Spearman Rho	*p* Value
Fibrosis LA	Estimated LA volume	Left-atrial structural remodeling	0.223	0.2545
Fibrosis LA	IVS thickness	Pressure-overload hypertrophic remodeling	0.130	0.5112
Fibrosis LA	Posterior wall thickness	Concentric remodeling	0.168	0.3928
Fibrosis LA	LVEDV	Ventricular chamber remodeling	0.147	0.4546
Fibrosis LA	LVEF	Systolic phenotype	−0.090	0.6500
Fibrosis LA	PASP	Pulmonary pressure burden	−0.191	0.3297
Fibrosis LA	Aortic mean gradient	Valvular pressure-overload severity	−0.008	0.9690
Fibrosis RA	PASP	Right-sided pressure burden	−0.028	0.8863
Fibrosis RA	Tricuspid peak gradient	Right-heart hemodynamic load	0.089	0.6542
Fibrosis RA	RV diameter	Right-sided chamber remodeling	0.012	0.9520
Fibrosis RA	Estimated LA volume	Interatrial remodeling link	0.395	0.0373
Fibrosis RA	Ascending aorta	Aortic-root/ascending-aorta remodeling phenotype	−0.424	0.0246
Fibrosis LA	Fibrosis RA	Interatrial histological fibrosis coupling	0.296	0.1257

**Table 6:** Exploratory regression models evaluating patient-level atrial fibrosis.

Model	Cohort	Dependent Variable	Main Predictor/Adjustment	B Coefficient (95% CI)	*p* Value; R^2^
Model 1	Whole cohort (n = 42)	Fibrosis LA	Severe AS status; adjusted for age and sex	8.548 (4.440 to 12.655)	*p* = 0.0001; R^2^ = 0.329
Model 2	Whole cohort (n = 42)	Fibrosis RA	Severe AS status; adjusted for age and sex	7.746 (4.396 to 11.097)	*p* < 0.0001; R^2^ = 0.386

## Data Availability

The data presented in this study are available from the corresponding author upon reasonable request, subject to institutional and ethical restrictions.
